# Blood Transcriptome Analysis of Septic Patients Reveals a Long Non-Coding *Alu*-RNA in the Complement C5a Receptor 1 Gene

**DOI:** 10.3390/ncrna8020024

**Published:** 2022-03-29

**Authors:** Åse Emblem, Erik Knutsen, Tor Erik Jørgensen, Hilde Fure, Steinar Daae Johansen, Ole-Lars Brekke, Tom Eirik Mollnes, Bård Ove Karlsen

**Affiliations:** 1 Research Laboratory and Department of Laboratory Medicine, Nordland Hospital Trust, 8005 Bodø, Norway; ase.eeg.emblem@nlsh.no (Å.E.); hifu33@gmail.com (H.F.); Ole.Lars.Brekke@nlsh.no (O.-L.B.); t.e.mollnes@medisin.uio.no (T.E.M.); 2 Department of Medical Biology, UiT The Arctic University of Norway, 9037 Tromsø, Norway; erik.knutsen@uit.no; 3 Genomics Division—FBA, Nord University, 8026 Bodø, Norway; tor.e.jorgensen@nord.no (T.E.J.); steinar.d.johansen@nord.no (S.D.J.); 4 Department of Clinical Medicine, UiT The Arctic University of Norway, 9037 Tromsø, Norway; 5 Centre of Molecular Inflammation Research, Norwegian University of Science and Technology, 7491 Trondheim, Norway; 6 Department of Immunology, Oslo University Hospital Rikshospitalet, University of Oslo, 0372 Oslo, Norway

**Keywords:** septicaemia, severe inflammation, *Alu* elements, *Alu*-lncRNAs, immune genes, complement receptors, RNA drug targets

## Abstract

Many severe inflammation conditions are complement-dependent with the complement component C5a-C5aR1 axis as an important driver. At the RNA level, the blood transcriptome undergoes programmed expression of coding and long non-coding RNAs to combat invading microorganisms. Understanding the expression of long non-coding RNAs containing *Alu* elements in inflammation is important for reconstructing cell fate trajectories leading to severe disease. We have assembled a pipeline for computation mining of new *Alu*-containing long non-coding RNAs by intersecting immune genes with known *Alu* coordinates in the human genome. By applying the pipeline to patient bulk RNA-seq data with sepsis, we found immune genes containing 48 *Alu* insertion as robust candidates for further study. Interestingly, 1 of the 48 candidates was located within the complement system receptor gene C5aR1 and holds promise as a target for RNA therapeutics.

## 1. Introduction

*Alu* elements are short interspersed transposable elements (SINEs) that have propagated in the primate lineage with more than one million copies and constitute approximately 10% of the human genome. *Alu* elements are dispersed throughout the genome with a bias toward gene-rich regions, concentrated in introns and untranslated regions (UTRs) of genes. Evolution has given rise to *Alu* subfamilies where *AluJ* and *AluS* represent the earliest evolved, and *AluY* the most recently evolved [1,2]. *Alu* elements are non-autonomous retrotransposons but may still transpose by hijacking enzymes expressed from genomic long interspersed transposable elements (LINEs) [3].

*Alu* elements are derived from the 7SL RNA and are therefore originally transcribed by RNA polymerase III (Pol III). As *Alu* elements have propagated through the primate genome, more than half of all known *Alu* elements are located within introns and UTRs of Pol II-transcribed genes, and it can be challenging to determine whether an *Alu* element is actively transcribed as an independent primary transcript (Pol III) or processed from a mRNA or a long non-coding RNA (lncRNA) Pol II transcript [4,5]. Pol III-transcribed *Alu* elements embedded within a Pol II transcriptional unit possess their own transcriptional start site (TSS) and regulatory mechanisms. Although transcriptional initiation can be correlated between Pol II and Pol III within a genomic region [6], the two polymerases have their individual initiators and pathways [7].

Transposable elements (TEs) are prevalent in lncRNAs, and many of the lncRNA functional domains are known to be derived from transposable elements [8] (reviewed in [9]). Natural antisense transcripts (NATs) are abundant in lncRNAs and may influence the expression of corresponding sense transcripts. The regulation can be expressed through RNA substitution editing, transcriptional interference, chromatin modifications, or interaction with protein complexes [10,11,12]. An example of *Alu*-dependent NAT regulation was presented by Carrier and co-workers [13]. Here, a NAT in the mouse transcriptome, called antisense *Uchl1*, was reported to regulate the protein synthesis of UCHL1 by base pairing at a post-transcriptional level. The activity of this antisense transcript required an embedded inverted SINEB2 element (the homolog to human *Alu* elements), which acts as a protein synthesis activation domain [13,14]. 

The regulatory mechanisms of *Alu*-RNA in gene expression reveal functional diversity. Freestanding Pol III-transcribed *Alu*-RNAs mainly impact gene regulation in *trans*, acting as *trans*-factors, repressing Pol II transcription, or in complex with ribonucleoproteins (RNPs) acting as translation initiators [6,15]. Pol III-transcribed *Alu*-RNAs are usually expressed at low levels due to the low efficiency of the *Alu*-promoters but are significantly increased during stress such as heat shock and virus infection. Here, *Alu*-RNA competes with 40S RNA for binding of the SRP9/14 complex of the signal recognition particle (SRP) and prevents the formation of stress granules [15,16]. 

Several reports have emphasised the functional role of *Alu* elements embedded in lncRNAs [9,13,17]. *Alu* elements embedded within introns can introduce alternative splice-sites in both pre-mRNA and lncRNA. Inverted repeat *Alu* (IR*Alus*) duplexes may lead to nuclear retention of mRNAs [18,19], and IR*Alus* may also be associated with the formation of circular RNA. *Alu* elements embedded in mRNA 3′ UTRs can interact with opposite oriented elements from a lncRNA, regulating target gene translation by sequence complementarity and creating IR*Alus* in *trans*, so-called Staufen1-mediated mRNA decay [20]. *Alu* elements embedded in 3′ UTRs can also bind miRNA [21]. *Alu*-RNAs can also function as structural scaffolds for protein complexes, hereunder binding DNA and RNP complexes and modulating transcription of specific genes or chromatin states [22]. Furthermore, *Alu*-RNAs can interact with the splicing machinery, influence the stability of mRNA, and activate or repress translation [23].

Genomic *Alu* elements have gained enhancer-like functions and are enriched for enhancer-like histone modifications in a tissue-specific manner [24]. *Alu*-RNAs transcribed from such enhancers have gained cell-specific enhancer functions to regulate the expression of nearby genes [5]. These enhancer RNAs (eRNAs) can influence the catalytic activity of chromatin modifier proteins, interact in enhancer-promoter looping, or act as traps for transcription factors [25,26].

The innate immune system consists of physical, chemical, and cellular defence mechanisms against pathogens. The complement system is a major component of the innate immune system and drives inflammation and cues leukocyte cellular differentiation in runaway infections such as septicaemia that cause tissue damage, organ failure, and substantial morbidity and mortality. The cascade consists of more than 40 proteins present in blood plasma or on cell surfaces, many of which have a well-characterised function [27,28]. The regulatory roles of lncRNAs in innate immunity have been increasingly studied (reviewed in [29]), but the roles of *Alu*-RNA in immune responses have not been clarified. 

Here we applied an *Alu*-lncRNA discovering pipeline to blood transcriptome data from septic patients and healthy controls [30]. Of 105 peripheral blood samples included in the study, 88 were used for downstream transcriptome analysis, 40 from healthy controls, and 48 from patients diagnosed with sepsis, septic shock, or severe infection. The findings were confirmed by a second sepsis cohort and our in-house RNA-seq data from an ex vivo whole blood model, where whole blood was activated with *Staphylococcus aureus*. Previous studies have shown that many intragenic and antisense lncRNAs regulate their immediate genomic neighbouring genes [31,32]. Thus, the aim of the present study was to systematically evaluate ~3500 canonical immune genes in order to predict and identify new putative *Alu*-containing lncRNAs in sepsis. A total of 48 *Alu* insertions were found in 26 immune genes, and by filtering for independent transcriptional start sites, 20 strong candidates were identified. By these criteria, we found the *C5aR1* receptor gene to contain a new *Alu*-lncRNA, which was further validated with RNA-seq data from the whole blood model.

## 2. Materials and Methods

**Figure 1:**
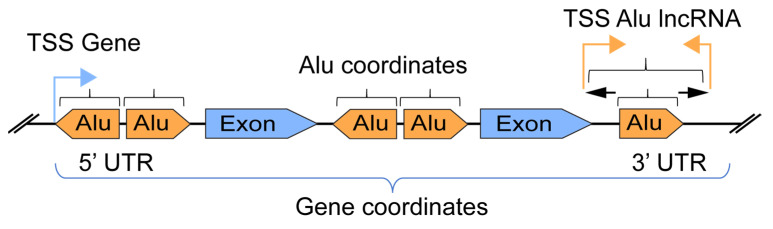
Overview of *Alu* elements versus gene coordinate definition and intersection.

We assembled an “*in house”* pipeline based on published genomic tools and databases for the identification of transcribed *Alu*-lncRNAs in immune gene coordinates. An overview of the *Alu*-lncRNA pipeline for setting the scope of the intersection between the gene and *Alu* elements is presented in Figure 1.

### 2.1. General Overview

UCSC table browser for *Alu* genomic coordinates:

Innatedb.com (accessed on 1 December 2021) [33] to download verified immune genes

HGNC to verify gene names and curate set: 

https://www.genenames.org/tools/multi-symbol-checker/ (accessed on 1 December 2021)

Ensembl biomart to get gene coordinates of curated gene names

Ensembl gene annotation: http://ftp.ensembl.org/pub/release-105/gtf/homo_sapiens/ (accessed on 1 December 2021)

Cage5 data for transcriptional start site (TSS) coordinates

https://fantom.gsc.riken.jp/5/ (accessed on 1 December 2021) [34]

Bedtools for the intersection of genomic coordinates

STAR aligner for unguided mapping of Fastq files

Homer, for quantification based on supplied *Alu* immune intersection coordinates

StrandNGS for Deseq, Hierarchical clustering

StrandNGS and R for UMAP statistics

### 2.2. External Bulk RNA-Seq Datasets

We selected 88 samples from a sepsis dataset from [30]: sepsis n = 18; septic shock n = 18; infection n = 12; healthy n = 40.

https://www.ncbi.nlm.nih.gov/bioproject?LinkName=sra_bioproject&from_uid=11431012 (accessed on 1 December 2021).

We also selected a dataset https://www.ncbi.nlm.nih.gov/bioproject/PRJNA607653/.

(accessed on 1 March 2022): Sepsis n = 18 and Healthy n= 18.

### 2.3. Whole Blood Model Induced with S. aureus

Whole blood from 6 healthy donors was collected after informed consent according to the guidelines from the regional ethics committee. All equipment, tips, and solutions were RNase and endotoxin-free. Fresh human whole blood was drawn into a PAXgene Blood RNA Tube (PreAnalytix, Hombrechtikon, Switzerland) as a baseline sample [35]. In addition, blood was drawn into 9 polypropylene tubes, 4.5 mL (Nunc Cryo Tubes, Thermo Fisher Scientific, Waltham, MA, USA), containing lepirudin (Refludan^®^, European Medicines Agency, Amsterdam, The Netherlands), final concentration 50 µg/mL. Aliquots of the lepirudin blood were divided into 4.5 mL Nunc cryotubes supplemented with phosphate-buffered saline (PBS) containing calcium and magnesium (Sigma Merck Life Science AS, Darmstadt, Germany) and preheated in a block heater at 37 °C for 5 min. Heat-inactivated *S. aureus* (1 × 10^8^ cells per mL) strain Cowan (ATCC 12598) or PBS was then added. Samples were incubated on a Rock’n roller at 37 °C for 0, 30, 60, and 120 min. The inflammatory reaction was stopped by the addition of EDTA (10 mM final concentration). Samples were centrifuged at 1500× *g* for 15 min at 4 °C. Plasma was removed and frozen at −80 °C. The plasma volume was replaced by PBS, and the blood tubes were briefly vortexed before adding a volume of Paxgene solution (2.73 mL per mL blood). Samples were stored at −80 °C for RNA isolation.

### 2.4. RNA Isolation for RNA-Seq

MagMAX™ for Stabilized Blood tubes RNA Isolation Kit (Ambion by Life Technologies, Carlsbad, CA, USA) was used for RNA extraction. The concentration and purity of isolated total RNA was measured using a NanoDrop 2000c spectrophotometer (Thermo Fisher Scientific, Waltham, MA, USA). RNA integrity number (RIN) was assessed by microfluidic capillary electrophoresis using an Agilent 2100 Bioanalyzer and the RNA 6000 Nano Chip Kit (Agilent Technologies, Santa Clara, CA, USA) RNA samples exhibited RNA integrity numbers (RIN) between 7.8 and 9.6 (mean RIN 8.8), indicating optimal RNA quality for downstream applications. RNA from the 6 donors was pooled based on concentration for RNA-seq and kept separate for other downstream experiments at −80 °C.

### 2.5. Illumina Sequencing

Next-generation sequencing data were prepared at Eurofins Genomics GmbH (Ebersberg, Germany) using commercially available chemistry. Total RNA was used as starting material; prior to library preparation, the rRNA was depleted using Ribo-Zero™ Magnetic Kit (Human/Mouse/Rat) (Illumina, San Diego, CA, USA). Subsequently, the library preparation was conducted using chemistry from New England Biolabs (NEBNext, Ipswich, MA, USA). All libraries were pooled and sequenced across 3 lanes on a HiSeq 2000 instrument with chemistry v3. The sequencing was performed using the original chemistry provided by Illumina with 100 bp paired-end reads. The sequencing data were included in this study to verify the whole blood model as an in vitro imitation of severe blood infections. The sequencing data are only presented here in Figure 3 and Table S4. This sequencing data is available on request.

### 2.6. Ion S5 Stranded RNA Sequencing

For the Ion S5 sequencing, the whole blood model was followed as described for the Illumina sequencing. One µg of total RNA was subjected to rRNA depletion using the Low Input RiboMinus™ Eukaryoute System v2 (Invitrogen, Thermo Fisher Scientific, Waltham, MA, USA). RNA libraries were constructed using Ion Total RNA-seq Kit v2 (Thermo Fisher Scientific, Waltham, MA, USA) according to the manufacturer’s protocol. Template preparation of libraries was carried out on the Ion Chef™ Instrument (Thermo Fisher Scientific, Waltham, MA, USA) and sequenced on Ion 540 chips using the Ion GeneStudio™ S5 System (Thermo Fisher Scientific, Waltham, MA, USA). The sequencing data were applied to this study in order to verify the transcriptional direction of the *Alu-RNAs* in Table 1 and are presented in Figure 3. The sequencing data are available on request.

### 2.7. Mapping of Transcripts

We downloaded the HG38 [13] without the alternative loci; this was used as a reference for the STAR mapper https://github.com/alexdobin/STAR (accessed on 1 December 2021)version STAR version = 2.7.9a with the following standard parameters [36]:

STAR --runThreadN 50 --runMode genomeGenerate --genomeDir/home//genomes/hg38 –genomeFastaFiles.

All datasets were quality checked for consistency with fastqc Version 0.11.9.

https://www.bioinformatics.babraham.ac.uk/projects/fastqc/

The reads were mapped unguided with no gtf coordinates with the following parameters:

STAR --readFilesCommand zcat --genomeDir/home/genomes/Hg38 --runMode alignReads --readFilesIn *R1_001.fastq.gz *R2_001.fastq.gz --runThreadN 50 --outSAMtype BAM Unsorted –outFileNamePrefix. 

The bam mapping files were sorted with samtools version 1.12 (htslib.org).

### 2.8. Alu Coordinates Intersection with Genes for Expression Analysis

For expression analysis of *Alu* insertion, we first downloaded the Repeatmasker track from the UCSC genome browser using the table browser tool. https://genome.ucsc.edu/ (accessed on 1 December 2021).

**Clade:** Mammal, **Genome:** Human, **Assembly:** Hg38, **Group:** Repeats, **Track:** RepeatMasker, **Region:** Genome and **Output:** Gtf. 

We parsed out 1,209,364 *Alu* coordinates from the Repeatmasker track, this Alu_only.gtf file was then converted to Alu_only.bed file with convert2bed from bedops toolset [37]. https://bedops.readthedocs.io/en/latest/index.html (accessed on 1 December 2021).

To intersect *Alu* coordinates with reference transcripts, we downloaded Homo_sapiens.GRCh38.105 from ensembl and used bedtools intersect [38] https://bedtools.readthedocs.io/en/latest/content/tools/intersect.html with the following parameters: ***bedtools intersect -a genes.bed -b alu.bed -wa -wb -f 0.009 > Alu_genes.bed*** that produced 993,146 intersections of *Alu* elements. This file was then converted back to a gtf file with the following script found on the bedtools webpage:

***awk ‘{print $1”\t”$7”\t”$8”\t”($2+1)”\t”$3”\t”$5”\t”$6”\t”$9”\t”(substr($0, index($0,$10)))}’ Alu_genes.bed > Alu_genes.gtf.***

### 2.9. Counting Alu Transcript Levels with the Homer Software

The Homer software (v4.11) [39] (http://homer.ucsd.edu/homer/) (accessed on 1 December 2021) is easy and configurable for implementing your own coordinates and reporting several statistical tests and normalisation of the data, such as VST (variance stabilization), Deseq, and raw reads. First, we performed the script ***makeTagDirectory*** to count all the reads for each sample bam file; we ran the following parameters to obtain transcriptome count data:

***analyzeRepeats.pl Alu_genes.gtf hg38 -strand both -min 400 -raw -dfile reads_to_analyze > out.csv.***

The ***out.csv*** was fed into Strand NGS software, ver 4. The conditions were set according to [30]. First, we carried out (uniform manifold approximation and projection) UMAP on all 1173 transcripts with the following parameters:

***Entity List: All Entities***
***Interpretation: condition1 (Non-averaged)***
***Distance Metric: Euclidean***
***Minimum Distance: 0.009999999776482582***
***Number of neighbours: 15***
***Initialization Method: Spectral***

Yang et al. found that UMAP was superior in clustering RNA-seq bulk data as compared with PCA and t-SNE algorithms [40]. We used Uwot version 0.1.11 (CRAN) and R version 4.1.0 (18 May 2021)—“Camp Pontanezen” according to the StrandNGS software options.

Then we produced a hierarchical cluster of 1173 transcripts with the following parameters:

***Created from Advanced Analysis operation: Clustering:***
***Entity List: All Entities***
***Interpretation: condition1 (Non-averaged)***
***Experiment: 22j***
***Clustering Algorithm: Hierarchical***
***Clustered By: Normalized intensity values***
***Clustered On: Entities and Conditions***
***Similarity Measure: Euclidean***
***Linkage Rule: Wards***
***Cluster Within Conditions: No***

The list of 1173 insertions were intersected with the Ensembl reference to represent 716 genes.

Filtering for robust and focused *Alu*-lncRNA candidates: in the Strand software, we filtered the results by a fold change of 1.3 and raw reads set to 1000 in one of the samples. This generated a list of 48 candidates that were manually inspected in IGV version 2.11 with the corresponding bam files. https://software.broadinstitute.org/software/igv (accessed on 1 December 2021).

All *p*-values were calculated by a two-way *t*-test and adjusted for false discovery rate by the Benjamini–Hochberg correction. Significant *p*-values were defined as <0.001.

## 3. Results

### 3.1. Alu Insertions in the Human Genome

Based on the dataset and software used in this study, we identified a total of 1.2 million *Alu* element insertions (UCSC) in the human genome (Hg38). These elements were divided into three families: *AluJ* (320,000 insertions), *AluS* (727,000 insertions), and *AluY* (149,000 insertions). The focus of this study was to investigate the intragenic *Alu* elements, and we found approximately 800,000 *Alu* insertions located within genes (coding and non-coding). We use the term “gene” as defined by ensembl.org—built from gene-wise alignments of the human proteome and alignments of human cDNAs. We analysed the positions of *Alu* elements within the gene bodies (Figure 1) and found that the most frequent *Alu* insertion sites were in introns (close to 90%, Figure S1). Less than 5% of *Alu* insertions were located in the 3′UTR. The subfamilies *AluJ*, *AluSx*, and *AluSx1* were the most common *Alu* insertions observed (Figure S1b).

### 3.2. Alu-RNAs in Blood from Sepsis Patients and Healthy Controls

**Figure 2:**
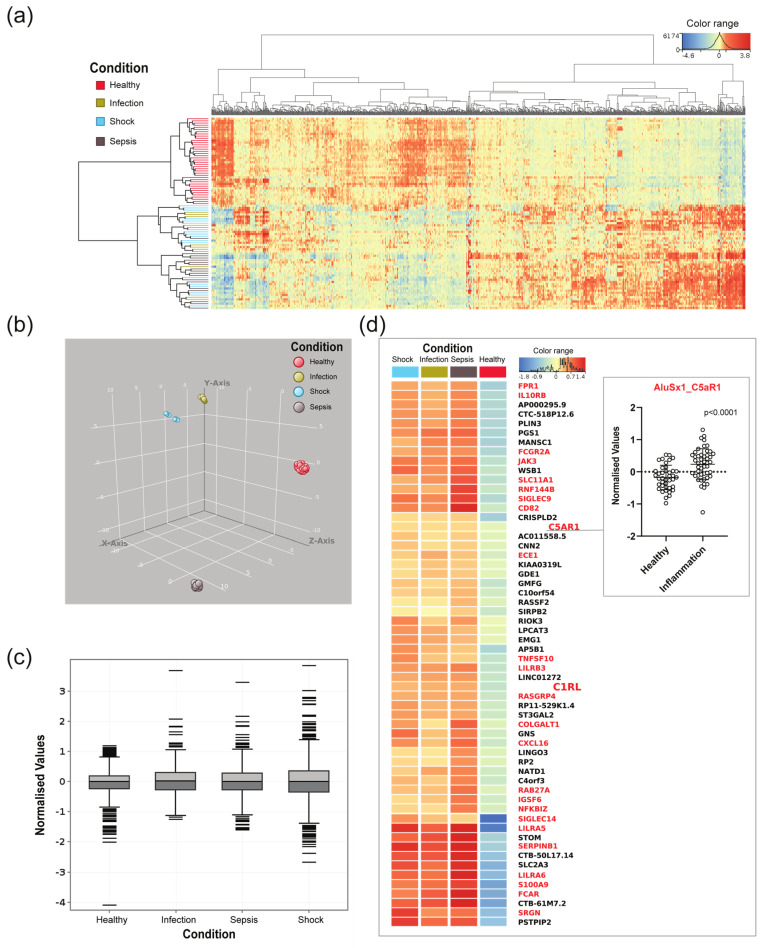
Expressed *Alu* elements transcribed from intragenic regions, from patients with infection, sepsis, septic shock, and healthy controls, displayed as hierarchical clustering and boxplot. (**a**) Heatmap produced in Strand NGS representing normalised values of intragenic *Alu*-containing transcripts, with a cut-off of 400 transcripts in at least one participant. This threshold produced 1173 *Alu* transcripts, represented in 716 genes. (**b**) UMAP supports clustering in Figure 1a, but in addition, shows that the dataset is separated between the conditions. (**c**) Boxplot showing normalised expression transcribed from intragenic regions and containing *Alu* elements. The boxes mark the medians and the upper and lower quartiles, and the whiskers extend to the most extreme values. (**d**) Heatmap of *Alu*-containing transcripts with the highest expression (>1000 raw reads in one or more samples) and highest fold change (threshold 1.3) between patients with inflammation and healthy controls. *Alu* transcripts from within the same genes are aggregated and produced transcripts from 58 genes that had at least one *Alu* insertion. Genes in red letters represent immune genes as defined by the Innatedb.com database (accessed on 1 December 2021). Box connected to *AluSx1* C5aR1 shows *p*-value of *p* ≤ 0.001.

Expressed *Alu* elements show high tissue specificity [5], and *Alu*-RNAs have been reported to be involved in cell differentiation and in responses to cellular stress, such as infection. We investigated the *Alu* element expression profile in the transcriptome of patients with severe inflammation. Transcriptome data from 12 patients with infection, 18 patients with sepsis, 18 patients with septic shock, and 40 healthy controls from the RNA-seq analysis of blood from sepsis patients and healthy controls—study (PRJNA647880) [30]—were included in this study (Table S1). The processed RNA-Seq data were filtered based on the following criteria: (1) Transcripts had to contain *Alu* elements in intragenic locations (excluding exons), and (2) the individual *Alu* element had to have a coverage of at least 400 transcripts in one or more of the participants. The Homer setting of 400 raw read cut-off generated 1173 *Alu* transcripts intersected in 726 genes, including 324 immune-related genes. Based on the 1173 transcripts, a hierarchical cluster heat map representing normalised values of expressed *Alu* elements in all participants was produced (Figure 2a, based on Table S2). We found that the *Alu*-containing transcripts were clustered based on health conditions, where the healthy controls and patients with inflammation constitute separate branches. About half of the transcripts showed an increased expression in healthy controls versus participants with inflammation, while a decrease in expression was observed in the other half. Although the hierarchical clustering separates healthy versus inflammation, the analysis did not differentiate between the inflammatory condition infection, sepsis, and septic shock. UMAP (Figure 2b), on the other hand, supported the hierarchical clustering, but in addition, it shows that the dataset was separated between all four conditions. Normalised expression values are also shown as box plots (Figure 2c); interestingly, the most extreme values were found in septic shock, the most severe form of sepsis.

The *Alu*-containing transcripts with the highest expression (>1000 raw reads in one or more samples) and highest fold change between patients with inflammation and healthy controls (fold change threshold 1.3) were considered the most interesting candidates with regard to a potential regulatory role. These criteria were met in 58 genes that had at least one *Alu* insertion (Figure 2d), and 26 of these (45%) were considered immune genes by the Innatedb.com database (accessed on 1 December 2021) (indicated by red letters in Figure 2d). Although not defined as immune genes by this definition, the remaining genes (black letters in Figure 2d) were to a large extent involved in transcriptional regulation, potentially leading to genome-wide changes in transcription and cell differentiation. A closer examination of the 48 *Alu* elements embedded in the immune genes from Figure 2d is presented in Table 1. Here we assessed genomic features such as location within the gene, *Alu* subfamily, adjacent TSS, and orientation of the *Alu* element relative to the gene in which they are inserted. TSS positions were of interest in order to identify standalone ncRNA transcripts. The TSS coordinates used in this study originate from CAGE data (Cap Analysis Gene Expression) from the FANTOM5 project. The TSS data were captured and aggregated from over 400 different human cell types using the CAGE protocol. In general, the CAGE protocol captures and links the 5′ CAP structure of a cells transcript, and by adding a linker to the tagged 3′ end, RNA-seq can be used to generate and map transcriptional start sites in the sample [41]. The *p*-values for the 1173 differentially expressed normalised *Alu* transcripts are given in Table S2. All differentially expressed normalised *Alu* transcripts in Table 1 were highly significant (*p* ≤ 0.001).

To further validate the robustness of our results, we applied a second RNA-seq dataset from peripheral blood mononuclear cells (PBMC) of sepsis patients, including healthy controls at Taizhou Hospital (PRJNA607653) (Table S1), which was analysed through the same pipeline (Figure S3, based on Table S3). Reproducing the biological results in different studies increases its validity and reliability of the data. We found a good correlation between the highly transcribed, differentially expressed *Alu*-RNAs. Of the 26 immune genes in Figure 2d, 15 genes were also included in the corresponding figure from the Taizhou dataset (Figure S3b).

We also introduced our own Illumina and Ion Torrent RNA-seq data from the whole blood model. This is a relatively uncomplicated in vitro model where we can measure how whole blood reacts to different stressors such as bacterial infection. We incubated blood from healthy donors with *Staphylococcus aureus* for two hours to mimic a severe bacterial infection. By obtaining similar data from the whole blood transcriptomes of sepsis patients and the bacteria induced ex vivo whole blood model, we strengthened the value of the model. Applying different sequencing platforms may illuminate variety in consistency between similar studies due to bias in the technologies. Illumina transcriptome data from the whole blood model were extracted based on a >200 reads threshold and intersected with *Alu*-RNA data from Table 1, obtaining transcripts from 16 *Alu* elements, nested in immune genes (Table S4). These results show that the in vitro whole blood model can to some extent mimic the expression in patient data.

Transcriptome data from the whole blood model sequenced by Ion Torrent were applied and manually inspected to verify the direction of the transcripts. All transcripts listed in Table 1 have the same orientation as the gene in which they are embedded. Interestingly, more than half of the transcribed *Alu* elements presented in Table 1 were found in the 3′UTR (27 in 3′UTR and 21 in introns), and most of the *Alu* elements with an associated TSS were also from a 3′UTR location. None of the *Alu* RNAs from Table 1 were located in the 5′UTRs. The differential expression and association with TSS suggested that these transcripts were 3′UTR-derived ncRNAs with regulatory functions in *cis* or *trans* and which have become less dependent on the processing of the host gene.

### 3.3. A Differentially Expressed Alu-Containing lncRNA Is Transcribed from the C5aR1 3′UTR

**Figure 3:**
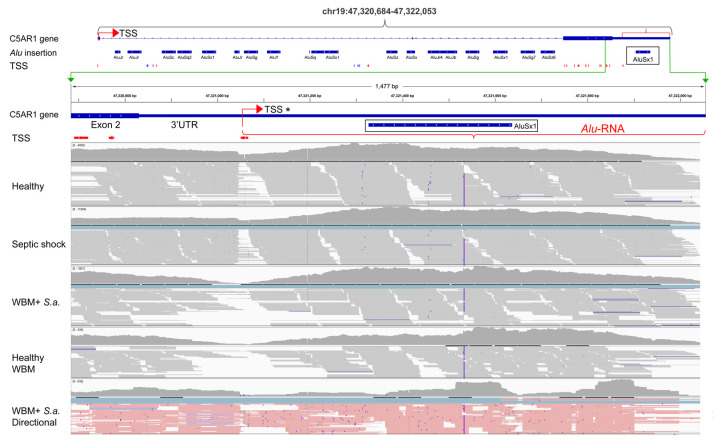
IGV presentation of the structure of the *C5aR1* gene with corresponding RNA-seq alignments. The top part shows the genomic structure of *C5aR1*, including CAGE-confirmed TSSs and *Alu* elements. A zoom-in on the 3′UTR beneath the green bracket includes alignments of RNA-seq data from the following samples: healthy, SRR12291428; septic shock, SRR12291512 (both from the Herwanto et al. study); WBM + *S.a.*, ex vivo whole blood model incubated with *S.aureus* for 120 min; Healthy WBM, ex vivo whole blood model incubated with phosphate-buffered saline (PBS) for 120 min; WBM + *S.a.* directional, ex vivo whole blood model incubated with *S. aureus* for 120 min, sequenced on Ion Torrent to generate directional reads (pink equals sense orientation). The TSS in the 3′UTR is marked as TSS*.

One interesting regulatory *Alu* element candidate was found in the 3′UTR of the complement component 5a receptor 1 (*C5aR1*) gene, also called cluster of differentiation 88 (*CD88*) gene. C5aR1 is a central 7-span G-coupled receptor in the complement cascade. Activated by the anaphylatoxin C5a, this receptor has a key role in the initiation and maintenance of several inflammatory responses in neutrophils and monocytes. We found that *AluSx1* located in the 3′UTR of the C5aR1 gene was highly and differentially expressed during inflammation compared with healthy controls (Figure 2d, right box and Table 1) and that this 3′UTR has an associated TSS located 300 nucleotides upstream of the *Alu* element (Figure 3, 3′UTR TSS marked as TSS*). This genomic feature has the potential of creating an *Alu* element containing lncRNA of about 1000 nucleotides (sequence in Figure S2). This finding was further supported by blasting the sequence in the NCBI EST database. We found two full-length 5′ enriched EST transcripts with overlapping sequences (AL571812.3 and AL572873.3) and several EST transcripts that cover the 3′part of the C5aR1-3′UTR-lncRNA including the *Alu* element (CA309875.1 and BP297702.1).

The *AluSx1* in the *C5aR1* 3′UTR was also found to be upregulated in the Taizhou study (Figure S3, Table S3) and our Illumina and Ion Torrent RNA seq data from the whole blood model. When we compared the *C5aR1* 3′UTR from the sepsis studies and the in vitro model; we observed an increase in transcription when comparing healthy control raw reads versus septic shock and when comparing raw reads from the whole blood model activated with bacteria versus healthy control. To investigate the independence of the *Alu*-lncRNA, we examined the RNA-Seq read mapping in the healthy and septic patients. In both cases, we saw a drop in read map alignment at the identified TSS (TSS*, Figure 3, representative patients included in the figure), which can give evidence towards the *Alu*-lncRNA as an independent transcript. These results were further supported by the Taizhou dataset (Figure S3).

## 4. Discussion

Delineation of the transcriptome landscape in septicaemia and severe inflammation is crucial for understanding the inflammation process in key primary immune cells such as neutrophils and monocytes. Whole blood as a tissue type is easily available for diagnostics and research, and molecular signatures of disease states can be discovered and assessed by proteomics and transcriptomics. In the current study, we report regulatory RNAs in the 3′ UTR of key genes involved in inflammation that contain *Alu* elements with robust expression patterns.

By applying a strict cut-off in the analysis, regarding read abundance and differential expression, we found robust *Alu*-RNA candidates belonging to the *AluS, AluJ*, and *AluY* families spanning intronic regions (n = 21) and 3′UTRs (n = 27). Both inverted and sense *Alu* elements (relative to nested gene and transcription) were observed. All expressed *Alu* elements were transcribed in the same orientation as the nested host gene. Eighteen of the *Alu* transcripts had a TSS located upstream (<1000 nucleotides) of the transcribed *Alu* element, while two *Alu* transcripts had a TSS in the 5′ part of the *Alu* element. From this total of twenty transcripts with associated TSSs, fourteen were from the 3′UTRs, and the remaining six were from intronic coordinates of immune genes. These findings were supported by addition of a second patient dataset (PRJNA607653) and the whole blood model using Illumina sequencing for paired-end and Ion Torrent for directional verification of transcripts. Differences in the datasets can be both biological and technical. RNA was isolated from patient whole blood, mononuclear cells, or whole blood drawn from healthy donors which were stimulated with bacteria in vitro. Different sequencing platforms with divergent library preparations will also affect the overall results. All these factors will influence the output of the analyses. We found a considerable reproducibility between the different datasets.

The distribution of *Alu* subfamilies is known to vary considerably across different transcript types. Kim and co-workers reported that *AluJ* subfamilies were overrepresented in lncRNAs, while *AluS* was more common in mRNA transcripts [8]. Furthermore, Su and co-workers put forward the idea that *Alu* elements may have evolved toward an enhancer function in the human genome, which were exemplified by old *Alu* subfamilies such as *AluSx*, *AluJo*, and *AluJb* [24]. These families were shown to have gained regulatory motifs, such as transcription factor binding sites and were enriched in histone post-translational modifications. We argue that *Alu*-containing transcripts in the immune genes represent regulatory RNA where the *Alu* element has gained a biological role. We found these *Alu* elements mainly from the subfamily *AluS* (n = 35, Table 1), and particularly from *AluSx*. This is consistent with the observation that *AluS* is overrepresented in mRNA transcripts. The role of *AluSx* as a transcriptional regulator is also reported by others. Cantarella and co-workers [42] showed that overexpression of *AluSx* in human fibroblast cell lines resulted in highly significant enrichment of pathways involved in cell cycle progression. This indicates a role as a transcriptional regulator of cell cycle genes. The regulatory role is also closely linked to the structural properties of *Alu*-RNA. *Alu*-RNAs have a defined folding, and the structure is key in interaction with proteins, DNA, and other RNAs. The structural importance of *Alu*-RNA, including *AluSx*, in regulation is emphasized in the study by Aune and co-workers [43], where the ability to activate innate immune responses resides on structural changes by A-to-I editing.

Several studies have investigated the differential expression of *Alu*-RNA in the brain during disease versus healthy state. Cheng et al. [44] showed how *Alu*-RNA regulated gene expression by binding RNA polymerase II and suppressing transcription in normal tissue. During stress, expression of *Alu*-RNA was upregulated, causing genome-wide transcriptional repression. This capacity to suppress RNA polymerase II is again regulated by *Alu*-RNA self-cleaving activity, generating another layer of regulation. This *Alu* RNA processing is increased in Alzheimer’s disease. Another study on Alzheimer’s disease found that the long non-coding RNA BC200 (*BCYRN1*) includes a left monomer *Alu* element. During normal aging the *BC200* RNA is reduced, while it is significantly upregulated in brain tissue of patients suffering from Alzheimer’s disease [45]. In brain tumours, the opposite effect is reported; Hwang and co-workers compared *Alu*-RNA expression between glioma brain tumour and normal tissues. They found downregulation of *Alu*-RNA in almost all pathologies of gliomas [46]. Common across all three studies was a change in expression between tissue from disease and healthy controls. The regulatory role of *Alu*-RNA is emphasized by the deregulation in disease.

*Alu* elements located in the 3′UTR of mRNA genes are known to be enriched in distinct cellular functions [47]. Liang and Yeh found that antisense *Alu* elements were common in 3′UTRs of gene transcripts associated with neurological and developmental processes, while sense transcripts were enriched in genes involved in immunological processes [44]. However, our observations of differentially expressed *Alu*-RNA embedded in immune genes (Table 1) appear to deviate from the findings by Liang and Yeh [44]. Most of our *Alu*-containing 3‘UTRs have an associated TSS, and we favour the idea that *Alu*-RNAs from 3′UTR transcripts are, at least partly, independent of their host mRNA transcripts. Interestingly, we found that the *AluSx1* element in *C5aR1* 3′ UTR had a clear TSS and indicates the formation of an *Alu*-lncRNA. Mapping of all datasets, both in vivo and *in vitro*, shows a scarcity of transcripts at the location of the TSS, suggestive of a separate 3′UTR transcript. The *AluSx1* element was highly and differentially expressed during inflammation compared with healthy conditions. The C5aR1 sits in a central hub of the complement inflammation cascade by directly binding C5a to induce inflammation in sepsis [35]. A major limitation of our work is the lack of functional studies validating the role of the *AluSx1* element as a transcriptional regulator. Such studies, including siRNA-mediated knockdown and luciferase reporter assays coupled with structural analysis, should be pursued in the future. However, the role of this group of *Alu*-containing ncRNA appears intriguing. They are differentially transcribed in immune cells during inflammation from an immune gene body, which suggests that these transcripts regulate either immunological or cell differentiation processes. Their role may include *cis*-regulation of the nested gene. Here, a mechanism such as transcription or translation initiation or repression, by binding DNA at promoter or enhancer sites, recruitment of regulatory proteins, or binding the mRNA directly, may be feasible.

*Alu* elements appear common within immune-related genes. In the search for new candidates for diagnostics and possible targets for RNA drugs such as gapmers and other RNA-mers [48], we found new transcripts containing *Alu* elements linked to canonical immune gene expression. Of particular interest is a new *Alu* element, *AluSx1* element, in the 3′ UTR of the complement component 5a receptor 1 (*C5aR1*) gene. Our study reveals the *C5aR1* 3′UTR as a promising target for studying the immune response in severe inflammation.

## Figures and Tables

**Table 1:** List of 48 *Alu* elements embedded in immune genes.

Gene Name	*Alu* Subfamily	Genomic Location	Position in Gene	TSS	*Alu* Orientation	*p*-Value
**ECE1**	*AluSx1*	chr1:21218585-21218890	3′UTR	no	Sense	4 × 10^−12^
**S100A9**	*AluSp*	chr1:153359680-153359988	Intron	no	Inverted	3 × 10^−33^
**FCGR2A**	*AluSz6*	chr1:161518618-161518922	3′UTR	yes ^1^	Inverted	2 × 10^−23^
**SRGN**	*AluSz*	chr10:69091596-69091908	Intron	no	Sense	9 × 10^−16^
**CD82**	*AluY*	chr11:44619480-44619789	3′UTR	yes	Sense	3 × 10^−13^
**C1RL**	*AluSc*	chr12:7095120-7095413	3’UTR	no	Sense	1 × 10^−21^
	*AluSq*	chr12:7097283-7097581	Intron	no	Sense	1 × 10^−14^
	*AluSq2*	chr12:7098913-7099254	Intron	yes ^1^	Inverted	6 × 10^−20^
**RAB27A**	*AluY*	chr15:55203412-55203721	3′UTR	no	Sense	1 × 10^−13^
**IGSF6**	*AluSx*	chr16:21639920-21640237	3′UTR	no	Sense	9 × 10^−8^
	*AluSx*	chr16:21640468-21640796	3′UTR	yes	Inverted	2 × 10^−10^
**CXCL16**	*AluSx3*	chr17:4733927-4734234	3′UTR	yes	Inverted	2 × 10^−12^
**COLGALT1**	*AluSz*	chr19:17581909-17582208	3′UTR	yes	Inverted	2 × 10^−11^
**JAK3**	*AluY*	chr19:17825619-17825921	3′UTR	yes	Inverted	1 × 10^−21^
	*AluSz*	chr19:17826103-17826414	3′UTR	yes	Inverted	1 × 10^−17^
	*AluSx*	chr19:17836278-17836571	Intron	no	Sense	3 × 10^−24^
**RASGRP4**	*AluSg*	chr19:38409458-38409752	3′UTR	no	Inverted	4 × 10^−17^
**C5AR1**	*AluSx1*	chr19:47321326-47321636	3′UTR	yes	Sense	2 × 10^−5^
**SIGLEC9**	*AluY*	chr19:51131305-51131590	Intron	no	Sense	2 × 10^−16^
	*AluSz*	chr19:51131634-51131933	Intron	no	Sense	2 × 10^−15^
	*AluSx1*	chr19:51133290-51133609	Intron	no	Sense	7 × 10^−19^
	*AluSc*	chr19:51134129-51134456	Intron	no	Inverted	4 × 10^−20^
**SIGLEC14**	*AluSx1*	chr19:51642741-51643045	3′UTR	yes	Inverted	4 × 10^−4^
**FPR1**	*AluSx1*	chr19:51745155-51745455	3′UTR	no	Sense	3 × 10^−20^
**LILRB3**	*AluJb*	chr19:54215999-54216282	3′UTR	no	Sense	1 × 10^−21^
	*AluY*	chr19:54216298-54216609	3′UTR	no	Sense	2 × 10^−21^
	*AluJo*	chr19:54216689-54216866	3′UTR	no	Sense	1 × 10^−20^
**LILRA6**	*AluSx3*	chr19:54238010-54238335	Intron	no	Sense	3 × 10^−19^
**LILRA5**	*AluSc*	chr19:54307138-54307296	3′UTR	no	Inverted	1 × 10^−26^
**FCAR**	*AluSx*	chr19:54889964-54890253	3′UTR	no	Inverted	4 × 10^−23^
	*AluSg*	chr19:54890415-54890727	3′UTR	yes	Inverted	6 × 10^−26^
**SLC11A1**	*AluSz*	chr2:218383482-218383779	Intron	no	Inverted	3 × 10^−13^
	*AluSx1*	chr2:218385379-218385690	Intron	no	Inverted	2 × 10^−8^
	*AluSc*	chr2:218388087-218388396	Intron	yes	Sense	3 × 10^−13^
	*AluSx3*	chr2:218388511-218388823	Intron	no	Sense	7 × 10^−20^
	*AluJb*	chr2:218388830-218389102	Intron	no	Sense	6 × 10^−19^
	*AluJo*	chr2:218389289-218389568	Intron	no	Sense	5 × 10^−14^
	*AluJo*	chr2:218390899-218391030	Intron	no	Sense	4 × 10^−14^
	*AluSx1*	chr2:218391611-218391922	Intron	yes	Inverted	4 × 10^−11^
	*AluSg*	chr2:218391923-218392055	Intron	yes	Inverted	2 × 10^−11^
	*AluSp*	chr2:218392056-218392362	Intron	yes	Inverted	3 × 10^−12^
	*AluJo*	chr2:218392363-218392634	Intron	yes	Inverted	4 × 10^−13^
	*AluSz6*	chr2:218395463-218395753	3′UTR	yes	Inverted	2 × 10^−17^
**IL10RB**	*AluSz*	chr21:33296666-33296973	3′UTR	no	Sense	8 × 10^−25^
**NFKBIZ**	*AluJo*	chr3:101860180-101860475	3′UTR	yes ^1^	Inverted	2 × 10^−12^
**TNFSF10**	*AluSc*	chr3:172505725-172506023	3′UTR	yes	Sense	1 × 10^−14^
**RNF144B**	*AluJo*	chr6:18467641-18467939	3′UTR	yes	Inverted	3 × 10^−16^
**SERPINB1**	*AluSx*	chr6:2832525-2832836	3′UTR	yes	Inverted	2 × 10^−21^

^1^ TSS is located in 5′ end of the *Alu* element.

## Data Availability

WBM S5 and Illumina data are available on request to the corresponding author: bard.ove.karlsen@nlsh.no.

## Supplementary Materials

The following supporting information can be downloaded at: https://www.mdpi.com/article/10.3390/ncrna8020024/s1, Figure S1: Genomic distribution of intragenic Alu elements, Figure S2: Gene sequence of the *Alu*-lncRNA transcript from the C5aR1 3′UTR, Figure S3: Expressed_Alu_elements_sepsis_healthy_Taizhou_study_Heatmap_IGV, Table S1: List of SRR external data included in this study, Table S2: 1173_Alu_insertions_patient_healthy_from_Homer_analysis, Table S3: 850_Alu_insertion_400_cutoff_Taizhou_study, Table S4: 16_Alu_insertions_200_cutoff_WBM_intersect_with_Table1.

## References

[1] Batzer M.A., Deininger P.L., Hellmann-Blumberg U., Jurka J., Labuda D., Rubin C.M., Schmid C.W., Zietkiewicz E., Zuckerkandl E. (1996). Standardized Nomenclature for Alu Repeats. J. Mol. Evol..

[2] Deininger P. (2011). Alu Elements: Know the SINEs. Genome Biol..

[3] Dewannieux M., Esnault C., Heidmann T. (2003). LINE-Mediated Retrotransposition of Marked Alu Sequences. Nat. Genet..

[4] Häsler J., Samuelsson T., Strub K. (2007). Useful “Junk”: Alu RNAs in the Human Transcriptome. Cell. Mol. Life Sci..

[5] Zhang X.-O., Gingeras T.R., Weng Z. (2019). Genome-Wide Analysis of Polymerase III-Transcribed Alu Elements Suggests Cell-Type-Specific Enhancer Function. Genome Res..

[6] Raha D., Wang Z., Moqtaderi Z., Wu L., Zhong G., Gerstein M., Struhl K., Snyder M. (2010). Close Association of RNA Polymerase II and Many Transcription Factors with Pol III Genes. Proc. Natl. Acad. Sci. USA.

[7] Jang K.L., Latchman D.S. (1989). HSV Infection Induces Increased Transcription of Alu Repeated Sequences by RNA Polymerase III. FEBS Lett..

[8] Kim E.Z., Wespiser A.R., Caffrey D.R. (2016). The Domain Structure and Distribution of Alu Elements in Long Noncoding RNAs and MRNAs. RNA.

[9] Fort V., Khelifi G., Hussein S.M.I. (2021). Long Non-Coding RNAs and Transposable Elements: A Functional Relationship. Biochim. Biophys. Acta Mol. Cell Res..

[10] Wight M., Werner A. (2013). The Functions of Natural Antisense Transcripts. Essays Biochem..

[11] Khorkova O., Myers A.J., Hsiao J., Wahlestedt C. (2014). Natural Antisense Transcripts. Hum. Mol. Genet..

[12] Zinad H.S., Natasya I., Werner A. (2017). Natural Antisense Transcripts at the Interface between Host Genome and Mobile Genetic Elements. Front. Microbiol..

[13] Carrieri C., Cimatti L., Biagioli M., Beugnet A., Zucchelli S., Fedele S., Pesce E., Ferrer I., Collavin L., Santoro C. (2012). Long Non-Coding Antisense RNA Controls Uchl1 Translation through an Embedded SINEB2 Repeat. Nature.

[14] Zucchelli S., Cotella D., Takahashi H., Carrieri C., Cimatti L., Fasolo F., Jones M.H., Sblattero D., Sanges R., Santoro C. (2015). SINEUPs: A New Class of Natural and Synthetic Antisense Long Non-Coding RNAs That Activate Translation. RNA Biol..

[15] Häsler J., Strub K. (2006). Alu RNP and Alu RNA Regulate Translation Initiation in Vitro. Nucleic Acids Res..

[16] Berger A., Ivanova E., Gareau C., Scherrer A., Mazroui R., Strub K. (2014). Direct Binding of the Alu Binding Protein Dimer SRP9/14 to 40S Ribosomal Subunits Promotes Stress Granule Formation and Is Regulated by Alu RNA. Nucleic Acids Res..

[17] Pérez-Molina R., Arzate-Mejía R.G., Ayala-Ortega E., Guerrero G., Meier K., Suaste-Olmos F., Recillas-Targa F. (2020). An Intronic Alu Element Attenuates the Transcription of a Long Non-Coding RNA in Human Cell Lines. Front. Genet..

[18] Prasanth K.V., Prasanth S.G., Xuan Z., Hearn S., Freier S.M., Bennett C.F., Zhang M.Q., Spector D.L. (2005). Regulating Gene Expression through RNA Nuclear Retention. Cell.

[19] Chen L.-L., DeCerbo J.N., Carmichael G.G. (2008). Alu Element-Mediated Gene Silencing. EMBO J..

[20] Gong C., Maquat L.E. (2011). LncRNAs Transactivate STAU1-Mediated MRNA Decay by Duplexing with 3′ UTRs via Alu Elements. Nature.

[21] Pandey R., Bhattacharya A., Bhardwaj V., Jha V., Mandal A.K., Mukerji M. (2016). Alu-MiRNA Interactions Modulate Transcript Isoform Diversity in Stress Response and Reveal Signatures of Positive Selection. Sci. Rep..

[22] West N., Roy-Engel A.M., Imataka H., Sonenberg N., Deininger P. (2002). Shared Protein Components of SINE RNPs. J. Mol. Biol..

[23] Chen L.-L., Yang L. (2017). ALUternative Regulation for Gene Expression. Trends Cell Biol..

[24] Su M., Han D., Boyd-Kirkup J., Yu X., Han J.-D.J. (2014). Evolution of Alu Elements toward Enhancers. Cell Rep..

[25] De Lara J.C.-F., Arzate-Mejía R.G., Recillas-Targa F. (2019). Enhancer RNAs: Insights Into Their Biological Role. Epigenet. Insights.

[26] Arnold P.R., Wells A.D., Li X.C. (2019). Diversity and Emerging Roles of Enhancer RNA in Regulation of Gene Expression and Cell Fate. Front. Cell Dev. Biol..

[27] Carroll M.V., Sim R.B. (2011). Complement in Health and Disease. Adv. Drug Deliv. Rev..

[28] Mollnes T.E., Huber-Lang M. (2020). Complement in Sepsis-When Science Meets Clinics. FEBS Lett..

[29] Walther K., Schulte L.N. (2021). The Role of LncRNAs in Innate Immunity and Inflammation. RNA Biol..

[30] Herwanto V., Tang B., Wang Y., Shojaei M., Nalos M., Shetty A., Lai K., McLean A.S., Schughart K. (2021). Blood Transcriptome Analysis of Patients with Uncomplicated Bacterial Infection and Sepsis. BMC Res. Notes.

[31] Mishra K., Kanduri C. (2019). Understanding Long Noncoding RNA and Chromatin Interactions: What We Know So Far. Noncoding RNA.

[32] Gil N., Ulitsky I. (2020). Regulation of Gene Expression by Cis-Acting Long Non-Coding RNAs. Nat. Rev. Genet..

[33] Breuer K., Foroushani A.K., Laird M.R., Chen C., Sribnaia A., Lo R., Winsor G.L., Hancock R.E.W., Brinkman F.S.L., Lynn D.J. (2013). InnateDB: Systems Biology of Innate Immunity and beyond--Recent Updates and Continuing Curation. Nucleic Acids Res..

[34] Lizio M., Harshbarger J., Shimoji H., Severin J., Kasukawa T., Sahin S., Abugessaisa I., Fukuda S., Hori F., Ishikawa-Kato S. (2015). Gateways to the FANTOM5 Promoter Level Mammalian Expression Atlas. Genome Biol..

[35] Mollnes T.E., Brekke O.-L., Fung M., Fure H., Christiansen D., Bergseth G., Videm V., Lappegård K.T., Köhl J., Lambris J.D. (2002). Essential Role of the C5a Receptor in E Coli-Induced Oxidative Burst and Phagocytosis Revealed by a Novel Lepirudin-Based Human Whole Blood Model of Inflammation. Blood.

[36] Dobin A., Davis C.A., Schlesinger F., Drenkow J., Zaleski C., Jha S., Batut P., Chaisson M., Gingeras T.R. (2013). STAR: Ultrafast Universal RNA-Seq Aligner. Bioinformatics.

[37] Neph S., Kuehn M.S., Reynolds A.P., Haugen E., Thurman R.E., Johnson A.K., Rynes E., Maurano M.T., Vierstra J., Thomas S. (2012). BEDOPS: High-Performance Genomic Feature Operations. Bioinformatics.

[38] Quinlan A.R., Hall I.M. (2010). BEDTools: A Flexible Suite of Utilities for Comparing Genomic Features. Bioinformatics.

[39] Heinz S., Benner C., Spann N., Bertolino E., Lin Y.C., Laslo P., Cheng J.X., Murre C., Singh H., Glass C.K. (2010). Simple Combinations of Lineage-Determining Transcription Factors Prime Cis-Regulatory Elements Required for Macrophage and B Cell Identities. Mol. Cell.

[40] Yang Y., Sun H., Zhang Y., Zhang T., Gong J., Wei Y., Duan Y.-G., Shu M., Yang Y., Wu D. (2021). Dimensionality Reduction by UMAP Reinforces Sample Heterogeneity Analysis in Bulk Transcriptomic Data. Cell Rep..

[41] Shiraki T., Kondo S., Katayama S., Waki K., Kasukawa T., Kawaji H., Kodzius R., Watahiki A., Nakamura M., Arakawa T. (2003). Cap Analysis Gene Expression for High-Throughput Analysis of Transcriptional Starting Point and Identification of Promoter Usage. Proc. Natl. Acad. Sci. USA.

[42] Cantarella S., Carnevali D., Morselli M., Conti A., Pellegrini M., Montanini B., Dieci G. (2019). Alu RNA Modulates the Expression of Cell Cycle Genes in Human Fibroblasts. Int. J. Mol. Sci..

[43] Aune T.M., Tossberg J.T., Heinrich R.M., Porter K.P., Crooke P.S. (2022). Alu RNA Structural Features Modulate Immune Cell Activation and A-to-I Editing of Alu RNAs Is Diminished in Human Inflammatory Bowel Disease. Front. Immunol..

[44] Cheng Y., Saville L., Gollen B., Veronesi A.A., Mohajerani M., Joseph J.T., Zovoilis A. (2021). Increased Alu RNA Processing in Alzheimer Brains Is Linked to Gene Expression Changes. EMBO Rep..

[45] Mus E., Hof P.R., Tiedge H. (2007). Dendritic BC200 RNA in Aging and in Alzheimer’s Disease. Proc. Natl. Acad. Sci. USA.

[46] Hwang T., Kim S., Chowdhury T., Yu H.J., Kim K.-M., Kang H., Won J.-K., Park S.-H., Shin J.H., Park C.-K. (2022). Genome-Wide Perturbations of Alu Expression and Alu-Associated Post-Transcriptional Regulations Distinguish Oligodendroglioma from Other Gliomas. Commun. Biol..

[47] Liang K.-H., Yeh C.-T. (2013). A Gene Expression Restriction Network Mediated by Sense and Antisense Alu Sequences Located on Protein-Coding Messenger RNAs. BMC Genom..

[48] Roberts T.C., Langer R., Wood M.J.A. (2020). Advances in Oligonucleotide Drug Delivery. Nat. Rev. Drug Discov..

