# Supplementary material for: Blood Transcriptome Analysis of Septic Patients Reveals a Long Non-Coding Alu-RNA in the Complement C5a Receptor 1 Gene
Source: Noncoding RNA. 2022 Mar 29;8(2):24. doi: 10.3390/ncrna8020024 (PMC9027897; doi:10.3390/ncrna8020024)
Supplement: Supplementary file 1 [file ncrna-08-00024-s001.zip › Supplementary_figures_S1-S3.pdf]

SUPPLEMENTARY FIGURES

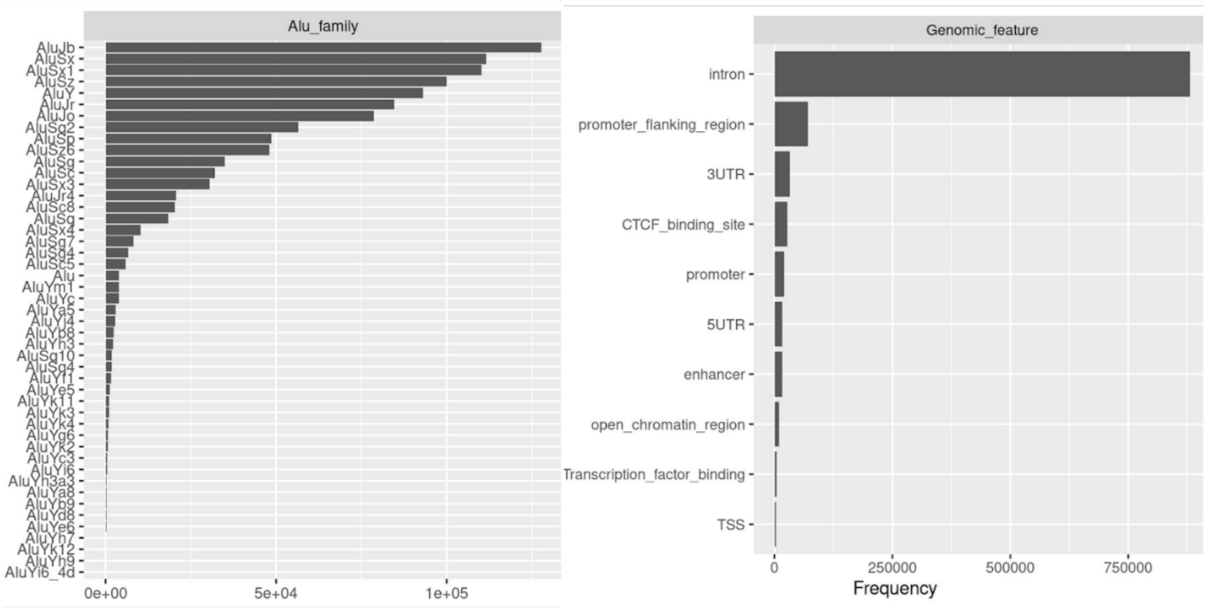

(**a**) (**b**)

Supplementary Figure S1. Genomic distribution of intragenic Alu elements. (**a**) Distribution of *Alu* subfamilies in all genes as defined by ensembl.org, and (**b**) position of *Alu* insertions in the gene body.

>C5aR1_3UTR_transcript

CCCCACCCCCCCCACACACACCATCTTTCCATCCCAGGCTTTTGAAAAACAAACAGAAACCCGTGTATCTGGGATATTTCCATATGGCAA
TAGGTGTGAACAGGGAACTCAGAATACAGACAAGTAGAAAGATTCTCGCTTAAAAAAAATGTATTTATTTTATGGCAAGTTGGAAAATAT
GTAACTGGAATCTCAAAAGTTCTTTGGGACAAAACAGAAGTCCATGGAGTTATCTAAGCTCTTGTAAGTGAGTTAATTTAAAAAAGAAAA
TTAGGCTGAGAGCAGTGGCTCACGCCTGTAATCCCAGAACTTTGGGAGGCTAAGGTGGGTGGATCACCTGAGGTCAAGAGTTCCAGACCA
GGCTGGCCAGCATGGTGAAACCCCGTCTGTACTAAAAATACAAAAAATTAACTGGGCATGGTAGTGGGTGCCTGTAATCCCAGCTACTTG
GGAGGCTGAGGTGGGAGAATTGCTCGAACTTGGAGGTGGAGGTTGTGGTGAGCCATGATCGCACCACTGCACTCTAGCCTGGGTGACCGA
GGGAGGCTCTGTCTCAAAAGCAAAGCAAAAACAAAAACAAAAACACCTAAAAAACCTGCAGTTTTGTTTGTACTTT**GTTTTTAAATTATG
CTTTCTATTTTGAGATCATTGCAAACTCAACACAATTGTAAGTAATGATACAGAGGGATCTTGTGTACCCTTCACCCAGCCTCCCCCAAT
GGCAACATCTTGCAAAACTACAATGTAGTCTCATAACCAGGATATTGACATTGATACAGTGAAGATACAGGACATTCTCATCACCACAGG
GATCCCCAGGATGCCCACTTCCCTCCACCCCCACACCCCAGCCGTGTCCCTAACCCCTGGCAACCAGGAATCCACTCTCCATTTCTATAA
TGTTGTCATTTCAAGAATGTTATTCAATGGAATCATATAGTATGTAACCTGTTTTGAGCTTAAAAAAAAAGTATA**CATGACTTTAATGAG
GAAAATAAAAATGAATATTGAAATGT

Supplementary Figure S2. Gene sequence of C5aR1 3’UTR transcript. *AluSx1* element in bold letters.

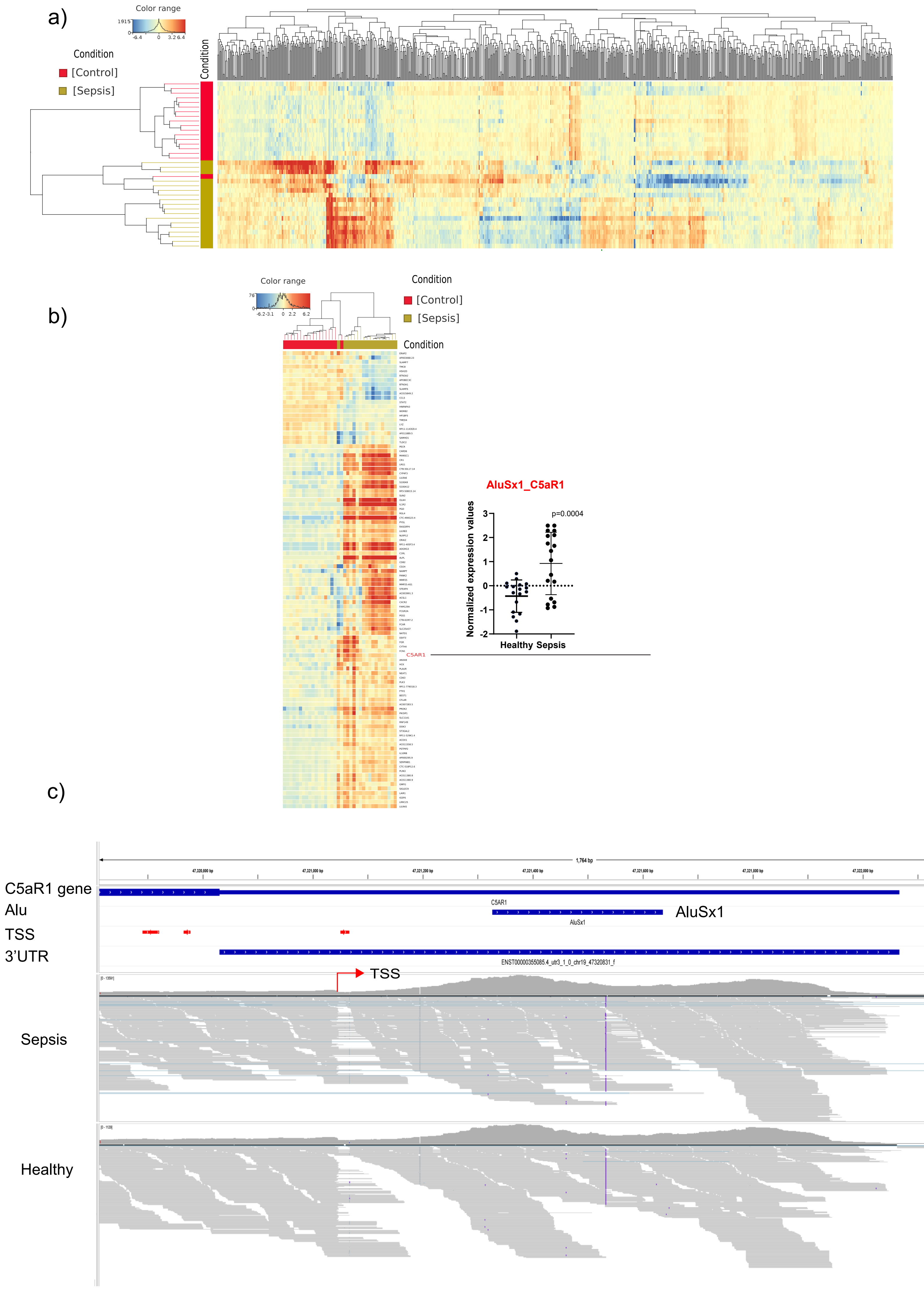

a)
Color range
Condition
[Control]
[Sepsis]
b)
Color range
Condition
[Control]
[Sepsis]
C5AR1
AluSx1_C5aR1
p=0.0004
Normalized expression values
Healthy
Sepsis
c)
1,764 bp
C5aR1 gene
Alu
TSS
3'UTR
C5AR1
AluSx1
AluSx1
ENST00000355085.4_utr3_1_0_chr19_47320831_f
TSS
Sepsis
Healthy

Figure S3. Expressed *Alu* elements transcribed from intragenic regions, RNA-seq dataset from peripheral blood mononuclear cells (PBMC) of sepsis patients including healthy controls at Taizhou Hospital (PRJNA607653). (**a**) Hierarchical clustering of normalised values of intragenic *Alu* containing transcripts, with a cut-off of 400 transcripts in at least one participant. This threshold produced 850 *Alu* transcripts (based on Table S3). (**b**) Heatmap of *Alu* containing transcripts with the highest expression (>1000 raw reads in one or more samples) and highest fold change (threshold 2.5) between patients with sepsis and healthy controls. *Alu* transcripts from within the same genes are. Line connected to *AluSx1* C5aR1 (red letters) shows *p*-value of $p \leq 0.004$. (**c**) IGV presentation of the structure of the C5aR1 gene with RNA-seq data from the Taizhou study. One alignment from sepsis patient (SRR11118030) and one heathy control (SRR11118016).
